# Four new species of the *Glyphiulus javanicus* group from southern China (Diplopoda, Spirostreptida, Cambalopsidae)

**DOI:** 10.3897/zookeys.741.23223

**Published:** 2018-03-07

**Authors:** Xuankong Jiang, Xuan Guo, Huiming Chen, Zhicai Xie

**Affiliations:** 1 CAS Key Laboratory of Aquatic Biodiversity and Conservation, Institute of Hydrobiology, Chinese Academy of Sciences, 7 Donghu South Rd., Wuhan 430072, Hubei, China; 2 Institute of Biology, Guizhou Academy of Sciences, 1 Longjiang Lane, Guiyang 550009, Guizhou, China; 3 University of Chinese Academy of Sciences, 19(A) Yuquan Rd., Beijing 100049, China

**Keywords:** Cave, China, *Glyphiulus*, millipede, new species, taxonomy

## Abstract

Hitherto, 24 species of the *Glyphiulus javanicus* group have been recorded, all endemic to Southeast Asia, including 14 in China. Nevertheless, this species group needs further exploration. In this context, four new species of this group are described, all collected from limestone caves in Southern China: *G. calceus* **sp. n.**, *G. foetidus* **sp. n.**, *G. guangnanensis* **sp. n.**, and *G. impletus* **sp. n.** They can be separated easily from each other and other congeners by their carinotaxic formulae, the structures of male legs I, and the gonopods. Due to the absence of any troglomorphic traits in our specimens, they may be troglophilic only.

## Introduction

Since Golovatch et al. (2007b) established and revised the *javanicus*-group, one of two groups of the species-rich millipede genus *Glyphiulus* Gervais, 1847, 25 valid species have been recorded up to date. They are endemic to southeast Asia, distributed from southern China, across Vietnam, Laos, and Thailand, to Java, Indonesia (Golovatch et al. 2007b, Jiang et al. 2017, Likhitrakarn et al. 2017). Among them, fourteen species occur in China, with most of them being cavernicolous:

*G. echinoides* Golovatch et al., 2011: from a cave in Fushui County, Guangxi Zhuang Autonomous Region;

*G. formosus* (Pocock, 1895): from Hong Kong, known only from female material;

*G. intermedius* Golovatch et al., 2007: from a cave in Chengdu County (possibly Chengdu City), Sichuan Province;

*G. latus* Jiang et al., 2017: from a cave in Muchuan County, Sichuan Province;

*G. liangshanensis* Jiang et al., 2017: from two caves in Liangshan Yi Autonomous Prefecture, Sichuan Province;

*G. obliteratoides* Golovatch et al., 2007: from three caves in Anshun County, Guizhou Province;

*G. obliteratus* Golovatch et al., 2007: from a cave in Mile County, Yunnan Province;

*G. paracostulifer* Golovatch et al., 2007: from a cave in Qianlin County, Guizhou Province;

*G. parobliteratus* Golovatch et al., 2007: from two caves in Suiyang County, Guizhou Province;

*G. pulcher* (Loksa, 1960): from a cave in Fulong Town, Daxin County, Guangxi Zhuang Autonomous Region;

*G. recticullus* Zhang & Li, 1982: from Qingyuan County, Zhejiang Province;

*G. sinensis* (Meng & Zhang, 1993): from a cave in Guanling County, Guizhou Province;

*G. subobliteratus* Golovatch et al., 2007: from a cave in Shilin County, Yunnan Province;

*G. zorzini* Mauriès & Nguyen Duy-Jacquemin, 1997: from a cave in Shuicheng County, Guizhou Province.

Recently, several taxonomical surveys of cave millipedes in southern China were carried out. As a result of these investigations, several species of *Glyphiulus* were identified, of which four new species of the *javanicus*-group are described here. Due to the absence of any troglomorphic traits in our specimens, they are thought to be troglophilic. Our findings confirm the hypothesis that southern China harbours an extremely high level of *Glyphiulus* diversity (Golovatch 2015).

## Materials and methods

Live specimens were collected by hand from localities in southern China. Type specimens are deposited in the Institute of Biology, Guizhou Academy of Sciences, Guiyang, China (**IBGAS**).

Live animals were first observed and photographed with a Canon EOS 5D Mark III camera with a Canon EF 100mm macro lens. All specimens were then preserved in 75 % ethanol. In the lab, some mature specimens were carefully picked out for examination, illustration, photography, and measuring using a Leica M205C stereomicroscope equipped with a Leica DFC450 Camera and LAS software (Version 4.1). Scanning electron micrographs (SEM) were taken with a Hitachi S-4800 field emission scanning electron microscope. Their geographical distributions were sketched with ArcGIS software (Ver. 10.2). All images were edited with Adobe Photoshop CC 2015 Software.

Terminology used in this paper follows the descriptions by Golovatch et al. (2007a, b, 2011) and Jiang et al. (2017).

## Taxonomy

### Order Spirostreptida Brandt, 1833

#### Family Cambalidae Cook, 1895

##### Genus *Glyphiulus* Gervais, 1847

###### Glyphiulus foetidus sp. n.

Taxon classificationAnimaliaSpirostreptidaCambalidae

http://zoobank.org/C306F018-1EAF-40C8-AC73-2208DF00FDA9

**Figure 1.**
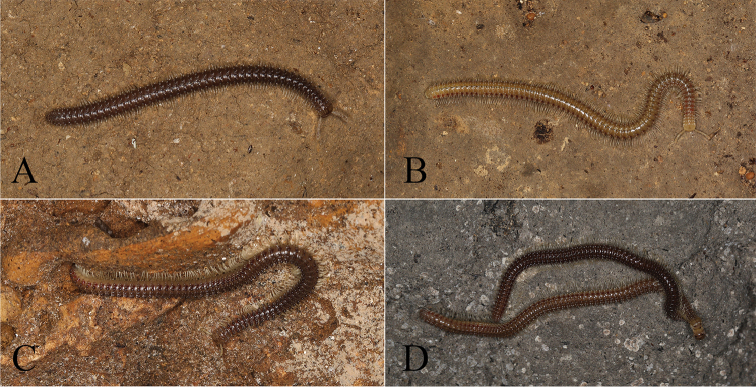
Living animals. **A** *Glyphiulus foetidus* sp. n. from Zhoubang Cave **B** *Glyphiulus calceus* sp. n. from Xianren Cave **C** *Glyphiulus guangnanensis* sp. n. from Miaopu Cave **D** *Glyphiulus impletus* sp. n. from Guanyin Cave.

**Figure 2.**
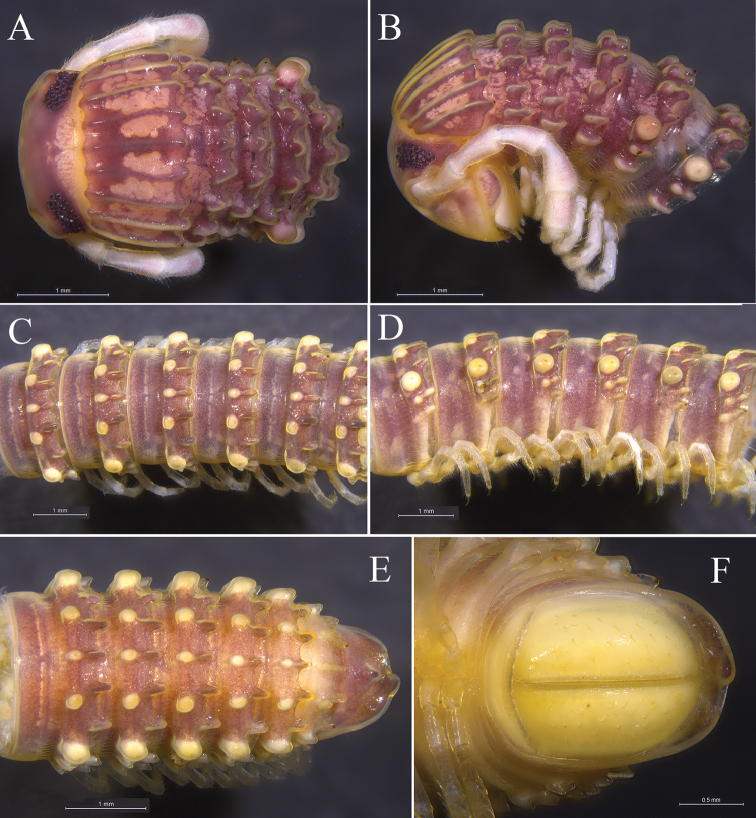
*Glyphiulus foetidus* sp. n., holotype. **A** anterior part of body, dorsal view **B** same, lateral view **C** midbody segments, dorsal view **D** same, lateral view **E** posterior part of body, dorsal view **F** same, ventral view.

**Figure 3.**
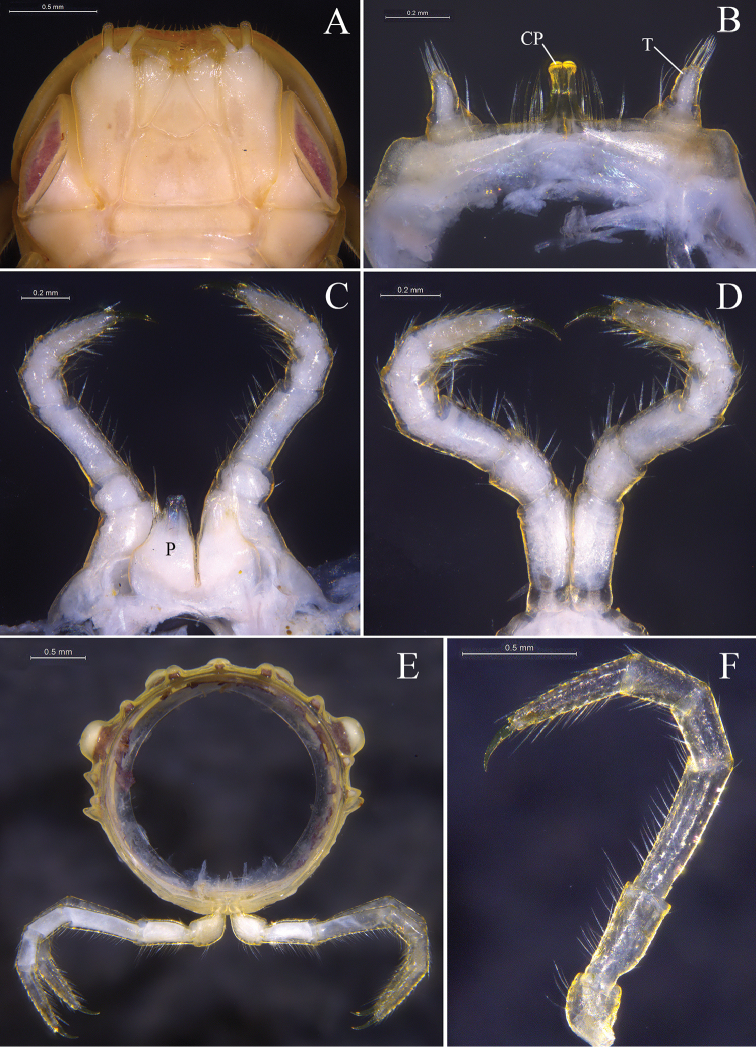
*Glyphiulus foetidus* sp. n., holotype. **A** gnathochilarium, ventral view **B** legs I, anterior view **C** legs II, caudal view **D** legs III, caudal view **E** cross-section of a midbody segment, caudal view **F** midbody leg, anterior view. Abbreviations: CP = coxal process; P = penes; T = telopodite.

**Figure 4.**
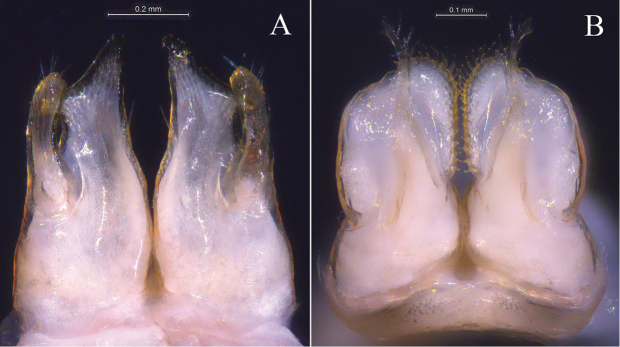
*Glyphiulus foetidus* sp. n., holotype. **A** anterior gonopods, caudal view **B** posterior gonopods, caudal view.

**Figure 5.**
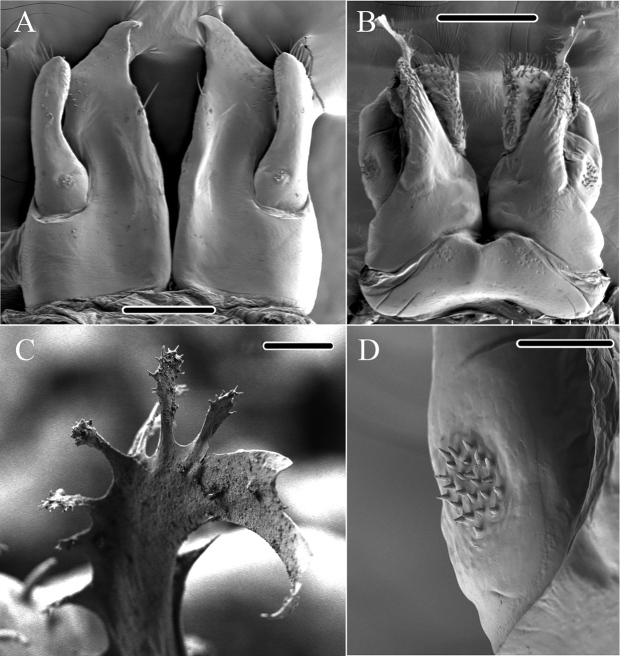
*Glyphiulus foetidus* sp. n., paratype. **A** anterior gonopods, caudal view **B** posterior gonopods, caudal view **C** flagellum of posterior gonopods **D** microsetae at lateral margin of posterior gonopods. Scale bars: **A, B** 0.2 mm **C, D** 0.02 mm.

**Figure 6.**
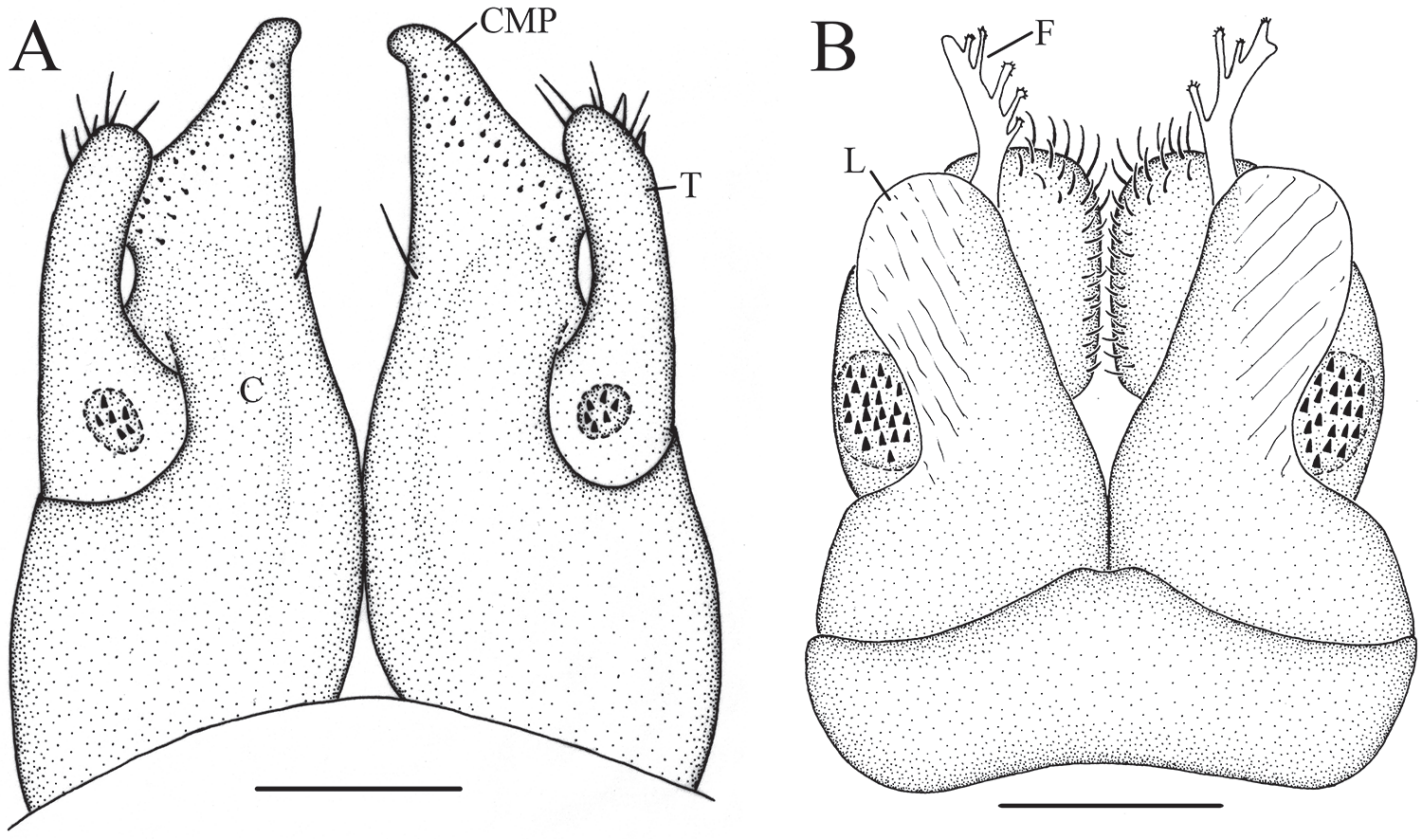
*Glyphiulus foetidus* sp. n., holotype. **A** anterior gonopods, caudal view **B** posterior gonopods, caudal view. Abbreviations: C = coxosternum; CMP = coxosternal mesal process; F = flagellum; L = lamelliform lobe; T = telopodite. Scale bars: **A, B** 0.2 mm.

Figs 1A , 2 , 3 , 4 , 5 , 6

####### Type material.

**Holotype** male, China: Guangxi Zhuang Autonomous Region, Xilin County, Zhoubang Village, Zhoubang Cave, 24°33.201'N, 105°06.634'E, alt. 820 m, 9 Jan. 2017, X.K. Jiang, H.M. Chen & X. Guo leg. (IBGAS). **Paratypes**: 61 males, 87 females and 12 juveniles, same date and locality as holotype (IBGAS).

####### Other material.

One male, Yunnan Province, Guangnan County, Bamei Town, Ake Village, Miaopu Cave, 24°14.767'N, 105°05.384'E, alt. 690 m, 8 Jan. 2017, X.K. Jiang, H.M. Chen & X. Guo leg. (IBGAS).

####### Etymology.

This specific name is derived from the Latin word *foetidus*, meaning ‘smelly’ and refers to the extremely strong and unpleasant smell of the animals.

####### Diagnosis.

The new species can be diagnosed by the following combination of morphological characteristics: (1) all crests on collum complete and fully developed, carinotaxic formula I–III + P + M; (2) telopodite of male legs I strongly degenerated, bi-segmented, as high as coxal process; (3) coxosternal mesal process of anterior gonopod prolonged and subtriangular; (4) flagellum of posterior gonopod short, with multiple branches at inner margin. See also Key below.

####### Description.

*Body* segments with 53–67p + 1–2a + T (holotype 67p + 1a + T). Body size of ca. 45–63 mm long and 2.3–3.0 mm wide (holotype 62 and 2.7 mm, respectively).

*Colouration*. Brown to dark brown *in vivo* (Fig. 1A). In fixed condition, head red-brown with yellow dapples; collum yellow-brown, anterior and posterior margins and the crests red-brown; midbody red-brown, lateral crests, ozoporiferous tubercles and anterior rows of metatergal crests light yellow; antennae and legs pale to light yellow (Fig. 2).

*Head*. Each eye patch with 30–45 pigmented ocelli arranged in five irregular vertical rows (Fig. 2A, B). Antennae slender, 2.88–3.35 mm long. Terminal part of antennomeres V expanded (Fig. 2B). Gnathochilarium with a separate promentum, polytrichous (Fig. 3A).

*Collum*. All crests on collum complete and fully developed, carinotaxic formula I–III + P + M (Fig. 2A, B).

*Body segments*. Postcollum constriction modest (Fig. 2A). Metatergal crests well-developed (Fig. 2A–E). Crests divided into two transverse rows of tubercles, carinotaxic formula 2/2+I/i+3/3+I/i+2/2. Anterior tubercle (except ozoporiferous one) small and round, posterior one strip-shaped (Fig. 2A–E). Ozoporiferous tubercles round, wider than high, obviously larger than other tubercles (Fig. 3E). Location of the tubercle behind ozopore relatively medial, set off from ozoporiferous tubercle (Figs 2C–E, 3E). Lateral crests rather small (Fig. 15). Midbody rings round in cross-section (Fig. 3E), 2.02–2.44 mm high (vertical diameter) and 2.15–2.56 mm wide (horizontal diameter), the ratio of height to width 0.92–0.97.

*Telson*. Epiproct simple, with a rounded caudal ridge and a strong dorsal tooth (Fig. 2E). Paraprocts convex, polytrichous. Hypoproct crescent-shaped (Fig. 2F).

*Walking legs*. Slender, 2.71–3.15 mm long, obviously longer than body width (Fig. 3E, F).

*Male sexual characters*. Male legs I strongly degenerated, with a pair of bi-segmented telopodites and a pair of large, subdigitiform, coxal processes. Coxal processes contiguous medially and curved forward, with clusters of long and robust setae at base (Fig. 3B). Male legs II normal. Penes trapeziform and small, each possessing three robust distolateral setae (Fig. 3C). Male legs III modified, with coxa especially slender and elongated (Fig. 3D). Femora VI and VII normal, not inflated.

*Anterior gonopods*. Coxosterna shield-like, sunken medially. Coxosternal mesal processes prolonged, obviously higher than telopodites. Telopodites one-segmented, placed laterally, curved and moveable, with several distal setae and a field of microsetae at base (Figs 4A, 5A, 6A).

*Posterior gonopods*. Compact (Figs 4B, 5B, 6B). Coxite with a medial lamelliform lobe and two rows of strong and curved setae at mediolateral margin. Flagella short with multiple branches at inner margin (Fig. 5C). Lateral margin with a field of microsetae (Fig. 5D).

####### Distribution.

Known only from the type locality, a cave in Xilin County, Guangxi, and another cave in Guangnan County, Yunnan. The two caves are ca. 35 kilometres apart.

###### Glyphiulus calceus sp. n.

Taxon classificationAnimaliaSpirostreptidaCambalidae

http://zoobank.org/39980A3D-3D10-4EFB-991D-A58E7AC13B54

**Figure 7.**
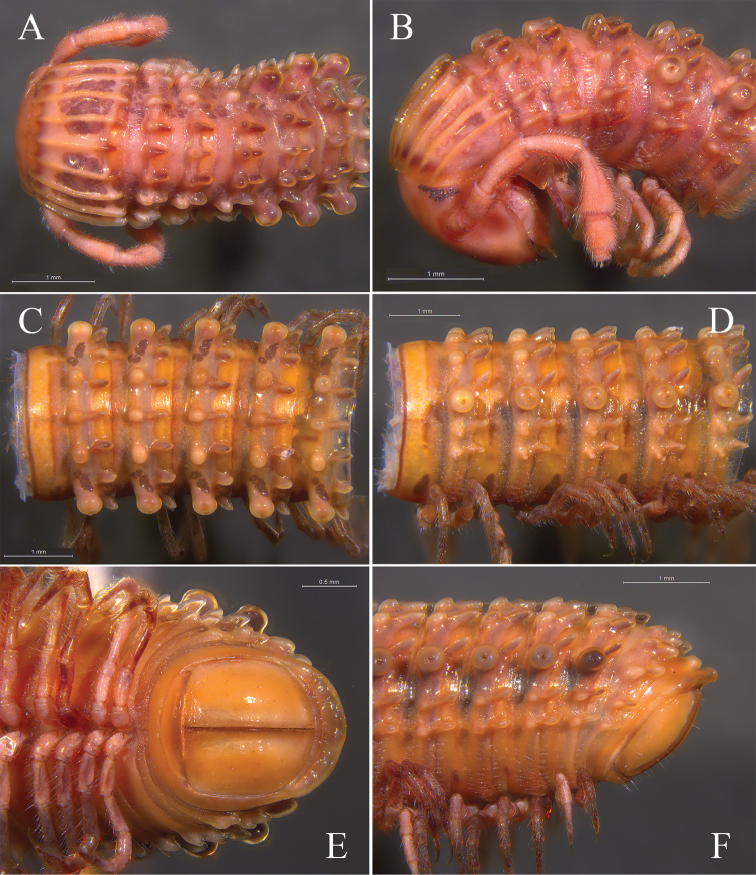
*Glyphiulus calceus* sp. n., holotype. **A** anterior part of body, dorsal view **B** same, lateral view **C** midbody segments, dorsal view **D** same, lateral view **E** posterior part of body, ventral view **F** same, lateral view.

**Figure 8.**
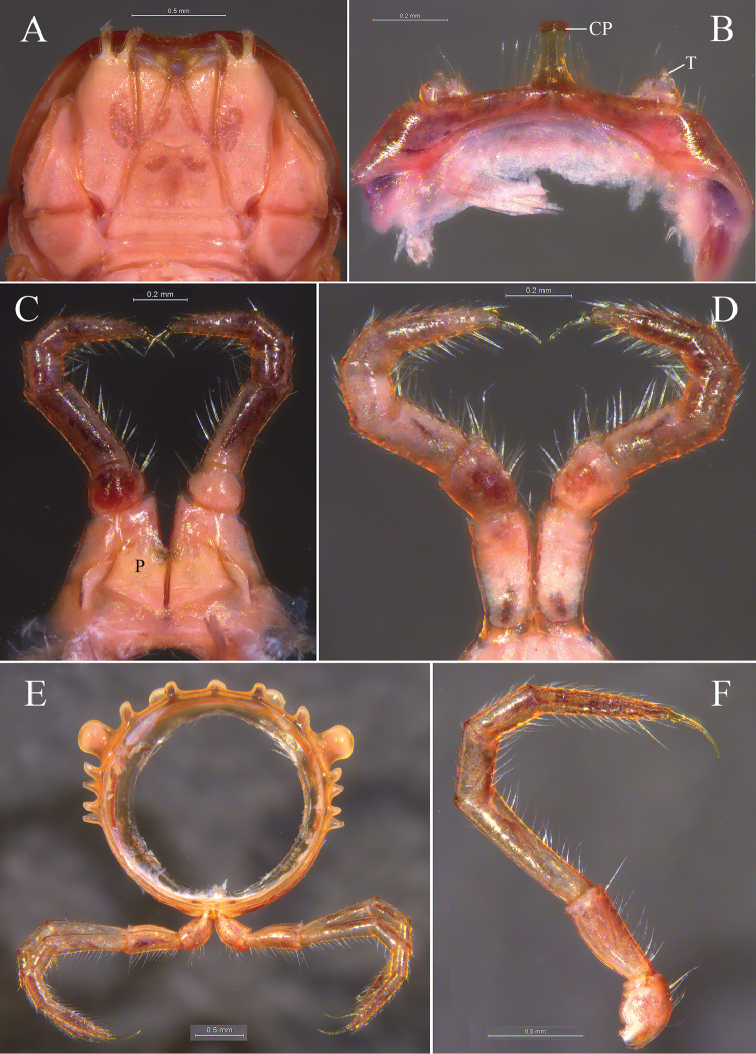
*Glyphiulus calceus* sp. n., holotype. **A** gnathochilarium, ventral view **B** legs I, anterior view **C** legs II, caudal view **D** legs III, caudal view **E** cross-section of a midbody segment, caudal view **F** midbody leg, anterior view. Abbreviations: CP = coxal process; P = penes; T = telopodite.

**Figure 9.**
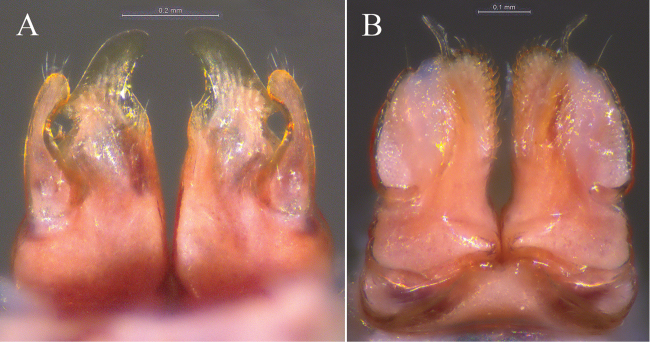
*Glyphiulus calceus* sp. n., holotype. **A** anterior gonopods, caudal view **B** posterior gonopods, caudal view.

**Figure 10.**
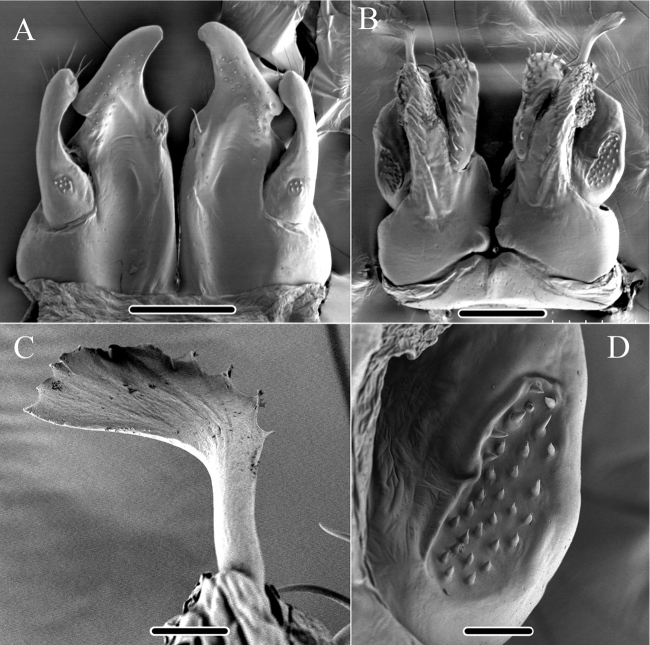
*Glyphiulus calceus* sp. n., paratype. **A** anterior gonopods, caudal view **B** posterior gonopods, caudal view **C** flagellum of posterior gonopods **D** microsetae at lateral margin of posterior gonopods. Scale bars: **A** 0.2 mm **B** 0.15 mm **C** 0.025 mm **D** 0.03 mm.

**Figure 11.**
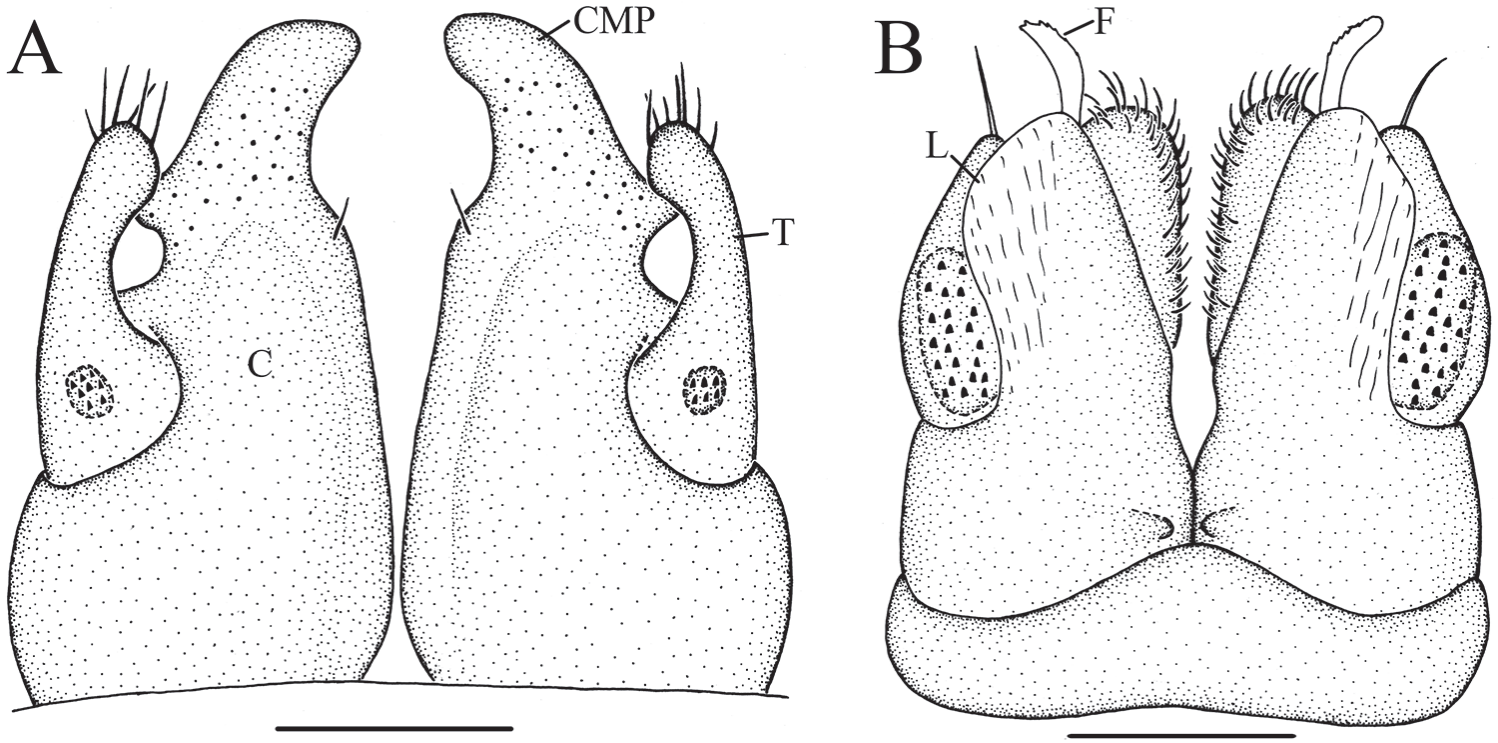
*Glyphiulus calceus* sp. n., holotype. **A** anterior gonopods, caudal view **B** posterior gonopods, caudal view. Abbreviations: C = coxosternum; CMP = coxosternal mesal process; F = flagellum; L = lamelliform lobe; T = telopodite. Scale bars: **A, B** 0.2 mm.

Figs 1B , 7 , 8 , 9 , 10 , 11

####### Type material.

**Holotype** male, China: Guangxi Zhuang Autonomous Region, Tian’e County, Bala Town, Madong Village, Hanyaotun, Xianren Cave 24°47.117'N, 107°04.851'E, alt. 900 m, 2 Jan. 2017, X.K. Jiang, H.M. Chen & X. Guo leg. (IBGAS). **Paratypes**: Thirteen males, 11 females and 1 juvenile, same date and locality as holotype (IBGAS).

####### Etymology.

This specific name is derived from the Latin word *calceus*, meaning ‘shoe’ and refers to the shape of the coxosternal mesal process of the anterior gonopod.

####### Diagnosis.

The new species can be diagnosed by the following combination of morphological characteristics: (1) all crests on collum complete and fully developed, carinotaxic formula I–III + P + M; (2) telopodite of male legs I bi-segmented, obviously shorter than coxal process; (3) coxosternal mesal process of anterior gonopod prolonged and shoe-shaped; (4) flagellum of posterior gonopod short and zigzag-shaped. See also Key below.

####### Description.

*Body* segments with 58–67p + 1–2a + T (holotype with 67p + 1a + T). Body size of ca. 45–63 mm long and 2.6–3.1 mm wide (holotype 58 and 2.9 mm, respectively).

*Colouration*. Brown to yellow brown *in vivo* (Fig. 1B); brown to red-brown in fixed condition (Fig. 7A–F).

*Head*. Each eye patch with 8–15 pigmented ocelli, arranged in two irregular vertical rows (Fig. 7B). Antennae slender, 2.90–3.28 mm long. Terminal part of antennomeres V expanded (Fig. 7B). Gnathochilarium with a separate promentum, polytrichous (Fig. 8A).

*Collum*. All crests complete and obvious, carinotaxic formula I–III + P + M (Fig. 7A, B).

*Body segments*. Postcollum constriction obvious (Fig. 7A). Metaterga strongly crested (Fig. 7A–F). Crests with two transverse rows of tubercles, carinotaxic formula 2/2+I/i+3/3+I/i+2/2. Anterior tubercle (except ozoporiferous one) small and upright, posterior one directed caudally, both with sharp tips (Fig. 7A–F). Ozoporiferous tubercle round, higher than broad, obviously larger than other tubercles (Fig. 8E). Location of the tubercle behind ozopore relatively medial, set off from ozoporiferous tubercle in caudal view (Figs 7C, D, 8E). Lateral crests rather small. Midbody rings round in cross-section (Fig. 8E), 2.10–2.48 mm high (vertical diameter) and 2.19–2.59 mm wide (horizontal diameter), the ratio of height to width 0.95–0.98.

*Telson*. Epiproct simple, with a rounded caudal ridge and a strong dorsal tooth. Paraprocts convex. Hypoproct crescent-shaped (Fig. 7E, F).

*Walking legs*. 3.17–3.67 mm long, obviously longer than body width (Fig. 8E, F).

*Male sexual characters*. Telopodite of male legs I strongly degenerated, bi-segmented. Coxal processes obviously longer than telopodites (Fig. 8B). Penes broad, tongue-shaped (Fig. 8C). Male legs III with slender and elongated coxa (Fig. 8D). Femora VI and VII normal, not inflated.

*Anterior gonopods*. Coxosternum shield-like, sunken medially. Coxosternal mesal processes of anterior gonopods elongated and shoe-shaped, obviously higher than telopodites. Telopodite one-segmented, curved and moveable, with round tip and a field of microsetae at base (Figs 9A, 10A, 11A).

*Posterior gonopods*. Mediolateral margins of coxite brush-like. Flagella short and zigzag-shaped (Fig. 10C). A long seta at anterolateral margin (Figs 9B, 11B). Lateral margin with a field of microsetae (Fig. 10D).

####### Distribution.

Known only from the type locality, a cave in Tian’e County, Guangxi Zhuang Autonomous Region.

###### Glyphiulus guangnanensis sp. n.

Taxon classificationAnimaliaSpirostreptidaCambalidae

http://zoobank.org/EDB6ECE1-6518-443A-8E64-882573FA9B9F

**Figure 12.**
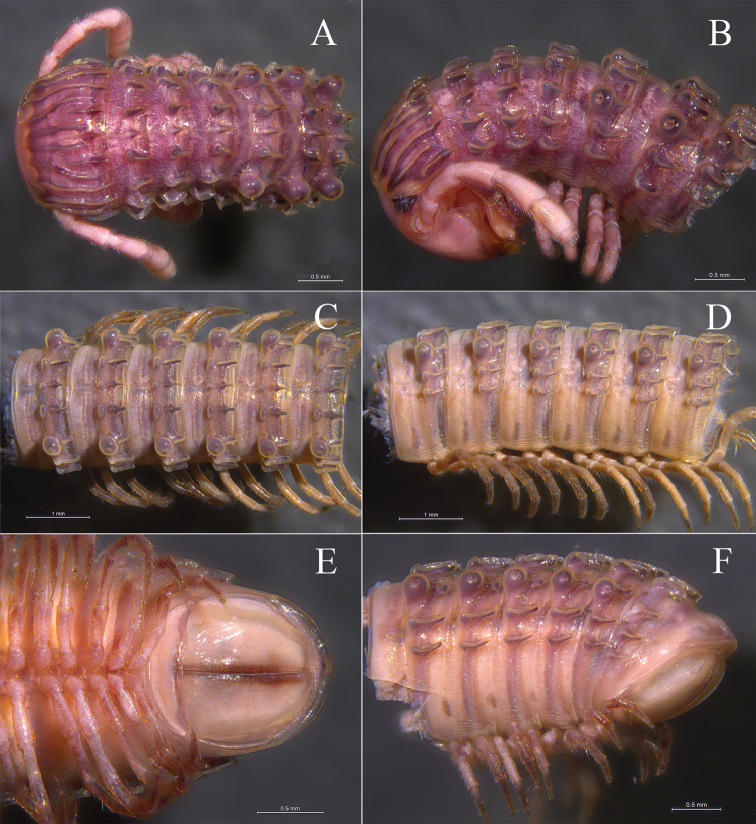
*Glyphiulus guangnanensis* sp. n., holotype. **A** anterior part of body, dorsal view **B** same, lateral view **C** midbody segments, dorsal view **D** same, lateral view **E** posterior part of body, ventral view **F** same, lateral view.

**Figure 13.**
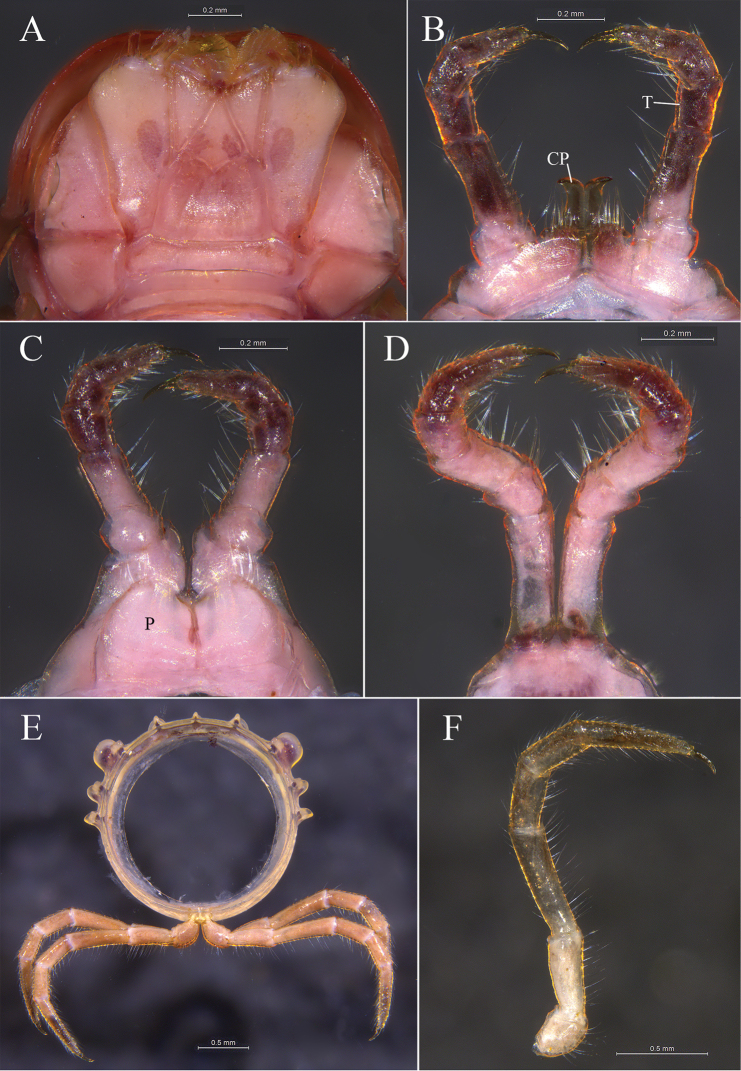
*Glyphiulus guangnanensis* sp. n., holotype. **A** gnathochilarium, ventral view **B** legs I, anterior view **C** legs II, caudal view **D** legs III, caudal view **E** cross-section of a midbody segment, caudal view **F** midbody leg, anterior view. Abbreviations: CP = coxal process; P = penes; T = telopodite.

**Figure 14.**
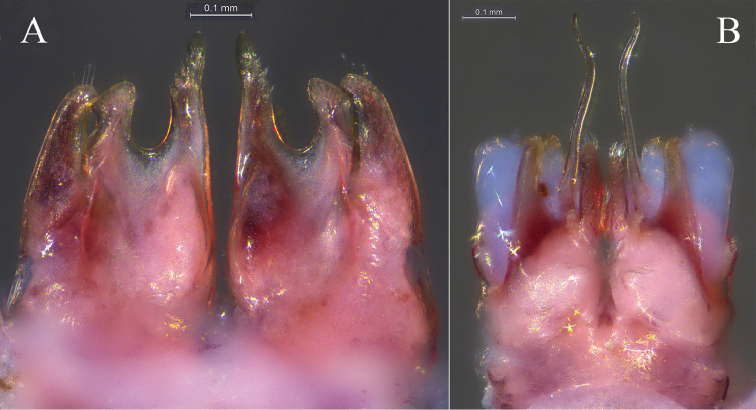
*Glyphiulus guangnanensis* sp. n., holotype. **A** anterior gonopods, caudal view **B** posterior gonopods, caudal view.

**Figure 15.**
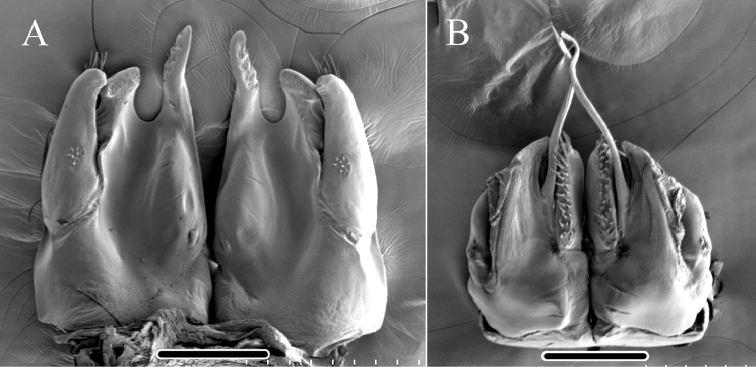
*Glyphiulus guangnanensis* sp. n., paratype. **A** anterior gonopods, caudal view **B** posterior gonopods, caudal view. Scale bars: **A, B** 0.2 mm.

**Figure 16.**
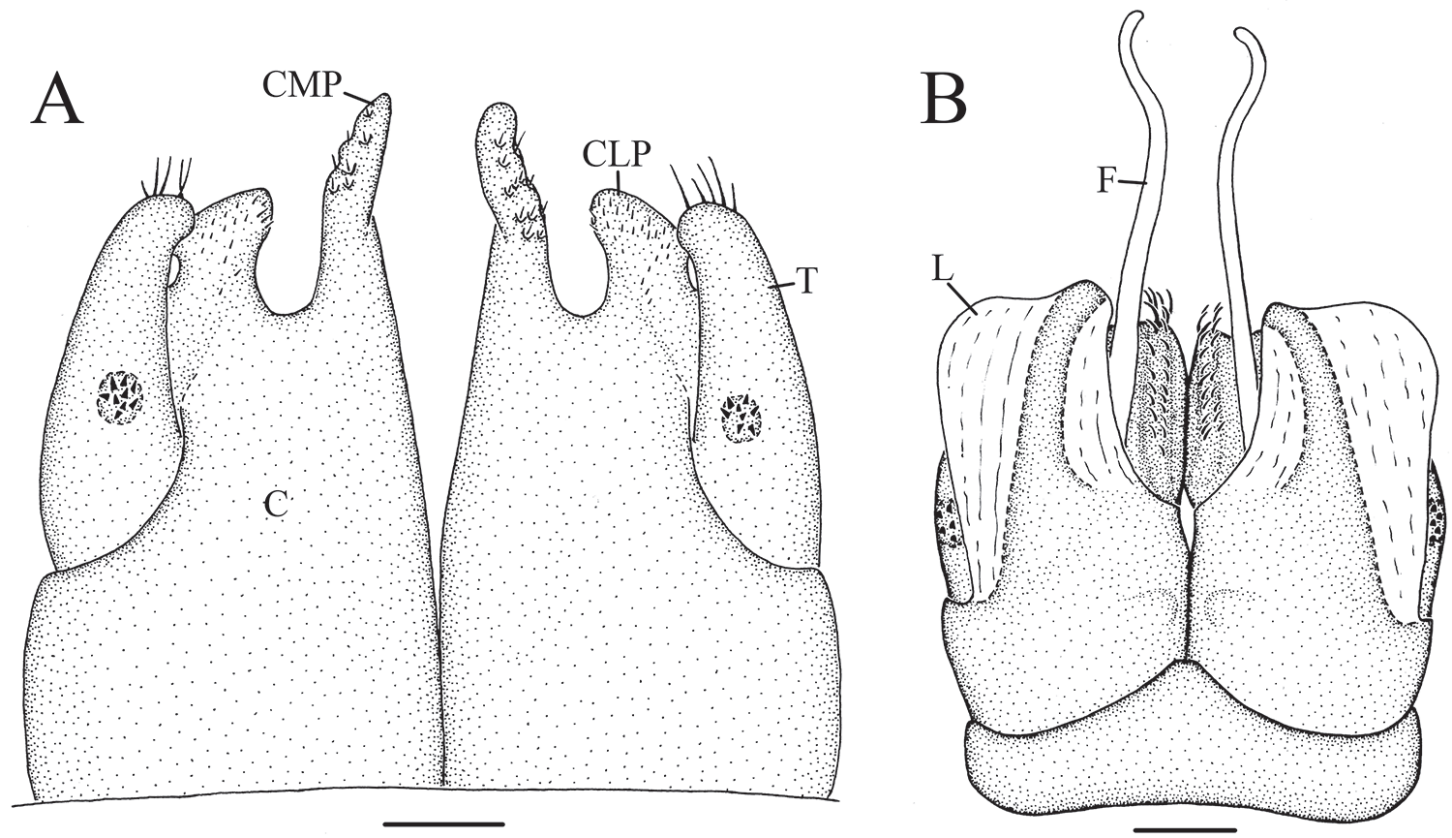
*Glyphiulus guangnanensis* sp. n., holotype. **A** anterior gonopods, caudal view **B** posterior gonopods, caudal view. Abbreviations: C = coxosternum; CLP = coxosternal lateral process; CMP = coxosternal mesal process; F = flagellum; L = lamelliform lobe; T = telopodite. Scale bars: **A, B** 0.1 mm.

Figs 1C , 12 , 13 , 14 , 15 , 16

####### Type material.

**Holotype** male, China: Yunnan Province, Guangnan County, Bamei Town, Ake Village, Miaopu Cave, 24°14.767'N, 105°05.384'E, alt. 690 m, 8 Jan. 2017, X.K. Jiang, H.M. Chen & X. Guo leg. (IBGAS). **Paratypes**: 9 males, 12 females and 9 juveniles, same date and locality as holotype (IBGAS).

####### Etymology.

This specific name is derived from the type locality.

####### Diagnosis.

The new species can be diagnosed by the following combination of morphological characteristics: (1) all crests on collum fully developed, carinotaxic formula 1a+2c+III–IV+5c+6a+pc+ma+pc+6a+5c+IV–III+2c+1a; (2) metatergal crests not divided, carinotaxic formula 2+I/i+3+I/i+2 (3) telopodite of male legs I complete, not degenerated, five-segmented; (4) anterior gonopod possessing a coxosternal mesal process and a coxosternal lateral process, coxosternal mesal process with a long and sharp tip, coxosternal lateral process with a blunt tip; (5) flagellum of posterior gonopod extremely long and smooth, slightly curved. See also Key below.

####### Description.

*Body* segments with 56–73p + 1a + T (holotype 73p + 1a + T). Body size of ca. 38–55 mm long and 2.0–2.3 mm wide (holotype 54 and 2.3 mm, respectively).

*Colouration*. Brown to dark brown *in vivo* (Fig. 1C); taupe to red-brown in fixed condition (Fig. 12A–F).

*Head*. Each eye patch with 9–12 pigmented ocelli arranged in 2–3 irregular vertical rows (Fig. 12B). Antennae slender, 2.20–2.38 mm long. Terminal part of antennomeres V slightly expanded (Fig. 12B). Gnathochilarium with a separate promentum, polytrichous (Fig. 13A).

*Collum*. All crests developed, carinotaxic formula 1a+2c+III–IV+5c+6a+pc+ma+pc+6a+5c+IV–III+2c+1a (Fig. 12A, B).

*Body segments*. Postcollum constriction modest (Fig. 12A). Metaterga strongly crested (Fig. 12A–F). All metatergal crests undivided (Fig. 12A–D, F), carinotaxic formula 2+I/i+3+I/i+2. Anterior part of crest round and broad, posterior part strip-shaped. Ozoporiferous tubercles large and round, as high as broad. Lateral crests fully developed. Midbody rings round in cross-section (Fig. 13E), 1.70–2.01 mm high (vertical diameter) and 1.74–2.08 mm wide (horizontal diameter), the ratio of height to width 0.96–0.99.

*Telson*. Epiproct with a rounded caudal ridge and an evident, axial, dorsal rib (Fig. 12F). Paraproct convex, with an evident depression near caudal edge, polytrichous. Hypoproct crescent-shaped (Fig. 12E, F).

*Walking legs*. 2.64–2.80 mm long, obviously longer than body width (Fig. 13E, F).

*Male sexual characters*. Telopodite of male legs I complete, five-segmented (Fig. 13B). Penes rather broad and round (Fig. 13C). Male legs II and III modified as usual (Fig. 13C, D). Femora VI and VII normal, not inflated.

*Anterior gonopods*. Coxosternum shield-like, sunken medially. Distal part of coxosternum with a deep indentation, the latter separating a mesal process and a lateral process. Coxosternal mesal process digitiform, obviously higher than telopodite. Coxosternal lateral process broad, with a blunt tip, nearly as high as telopodite. Telopodite short, one-segmented with thin and round tip and a field of microsetae at base (Figs 14A, 15A, 16A).

*Posterior gonopods*. Mediolateral margins of coxite brush-like. Flagella smooth, curved and extremely long. Lateral margin with a field of microsetae (Figs 14B, 15B, 16B).

####### Distribution.

Known only from the type locality, a cave in Guangnan County, Yunnan Province.

####### Notes.

Since the definitions of *Glyphiulus* and *Hypocambala* are still uncertain, this new species may be a member of *Hypocambala*. Mauriès (1977) considered that the two genera are distinguished only by the absence (*Hypocambala*) and presence (*Glyphiulus*) of transverse crests on body. Golovatch et al. (2011) dealt with the crests as a species-level character, and transferred *Glyphiulus vietnamicus* Mauriès, 1977 to *Hypocambala* based on the complete male legs I. However, this arrangement didn’t fully resolve this problem. In the genus *Glyphiulus*, there are still several species which present the same feature of male legs I and were not transferred to *Hypocambala*, for example *G. costulifer*, *G. intermedius*, *G. parobliteratus*, *G. percostulifer*, *G. pulcher*, and *G. semicostulifer*. A serious revision of the two genera is definitely needed but until then, this new species is assigned to *Glyphiulus*.

Usually, one cave supports one species of Cambalopsidae (Likhitrakarn et al. 2017). However, in our investigations, it was found that two species (*G. guangnanensis* sp. n. and *G. foetidus* sp. n.) could coexist in one place (Miaopu Cave), possibly due to the fact that they are troglophilic. Besides this, sympatry is also true for *G. semigranulatus* (likely troglophilic) and *G. obliteratus* (presumably troglobitic) which coexist in another cave (Bailong Cave).

###### Glyphiulus impletus sp. n.

Taxon classificationAnimaliaSpirostreptidaCambalidae

http://zoobank.org/F02E3546-7C94-4C0E-8091-88D4999003B4

**Figure 17.**
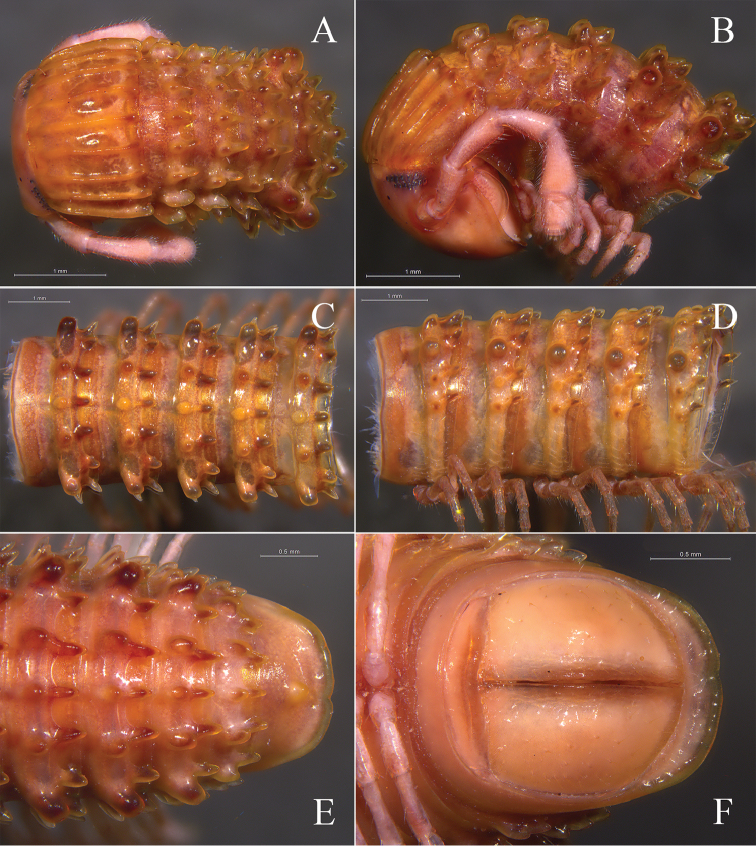
*Glyphiulus impletus* sp. n., holotype. **A** anterior part of body, dorsal view **B** same, lateral view **C** midbody segments, dorsal view **D** same, lateral view **E** posterior part of body, dorsal view **F** same, ventral view.

**Figure 18.**
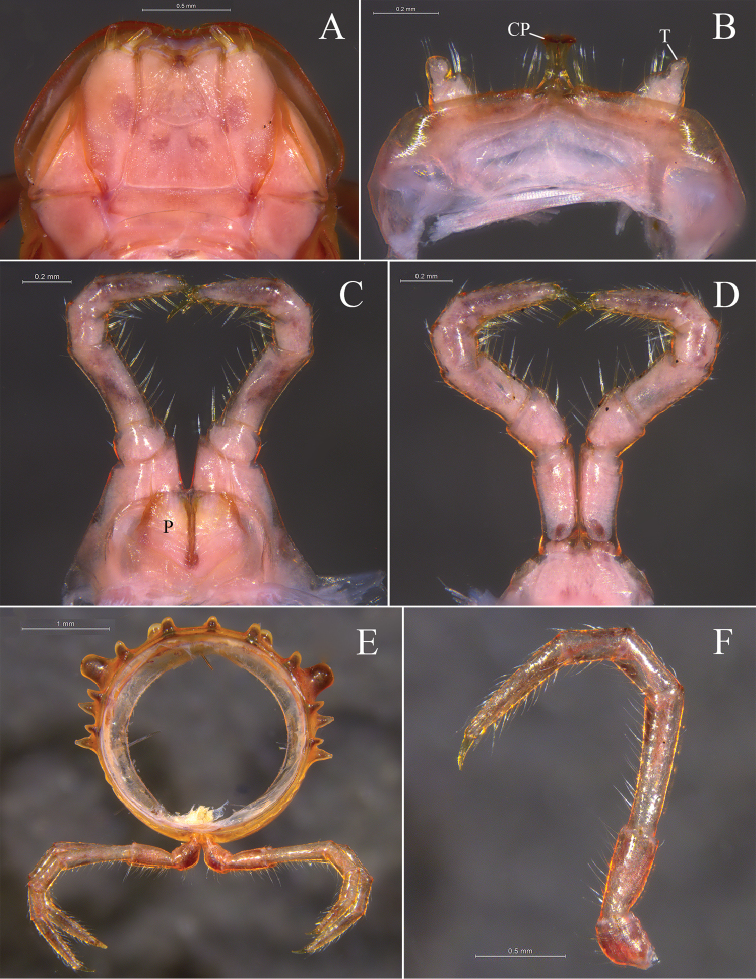
*Glyphiulus impletus* sp. n., holotype. **A** gnathochilarium, ventral view **B** legs I, anterior view **C** legs II, caudal view **D** legs III, caudal view **E** cross-section of a midbody segment, caudal view **F** midbody leg, anterior view. Abbreviations: CP = coxal process; P = penes; T = telopodite.

**Figure 19.**
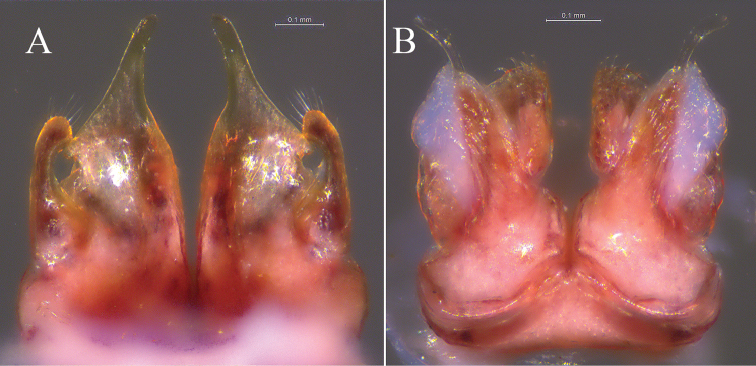
*Glyphiulus impletus* sp. n., holotype. **A** anterior gonopods, caudal view **B** posterior gonopods, caudal view.

**Figure 20.**
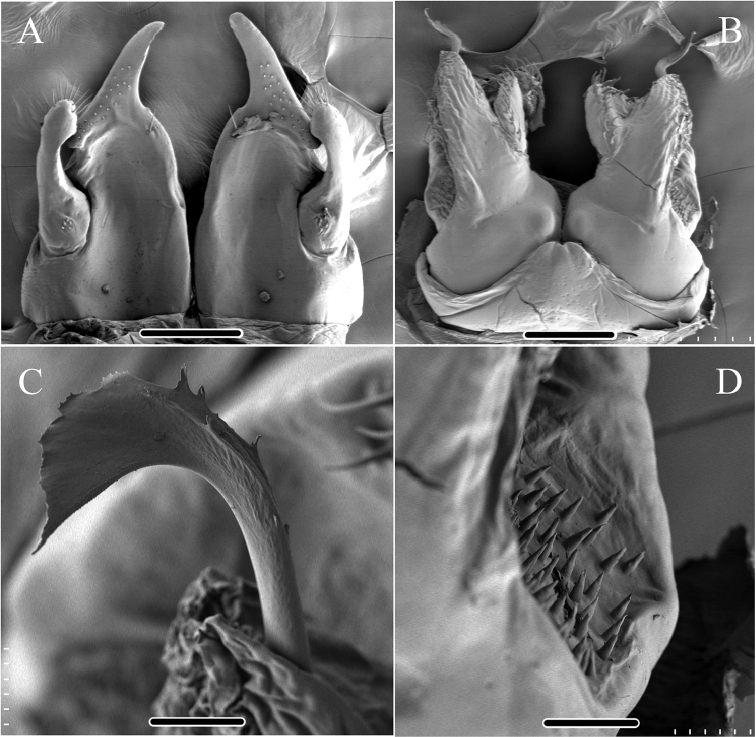
*Glyphiulus impletus* sp. n., paratype. **A** anterior gonopods, caudal view **B** posterior gonopods, caudal view **C** flagellum of posterior gonopods **D** microsetae at lateral margin of posterior gonopods. Scale bars: **A** 0.2 mm **B** 0.15 mm **C, D** 0.3 mm.

**Figure 21.**
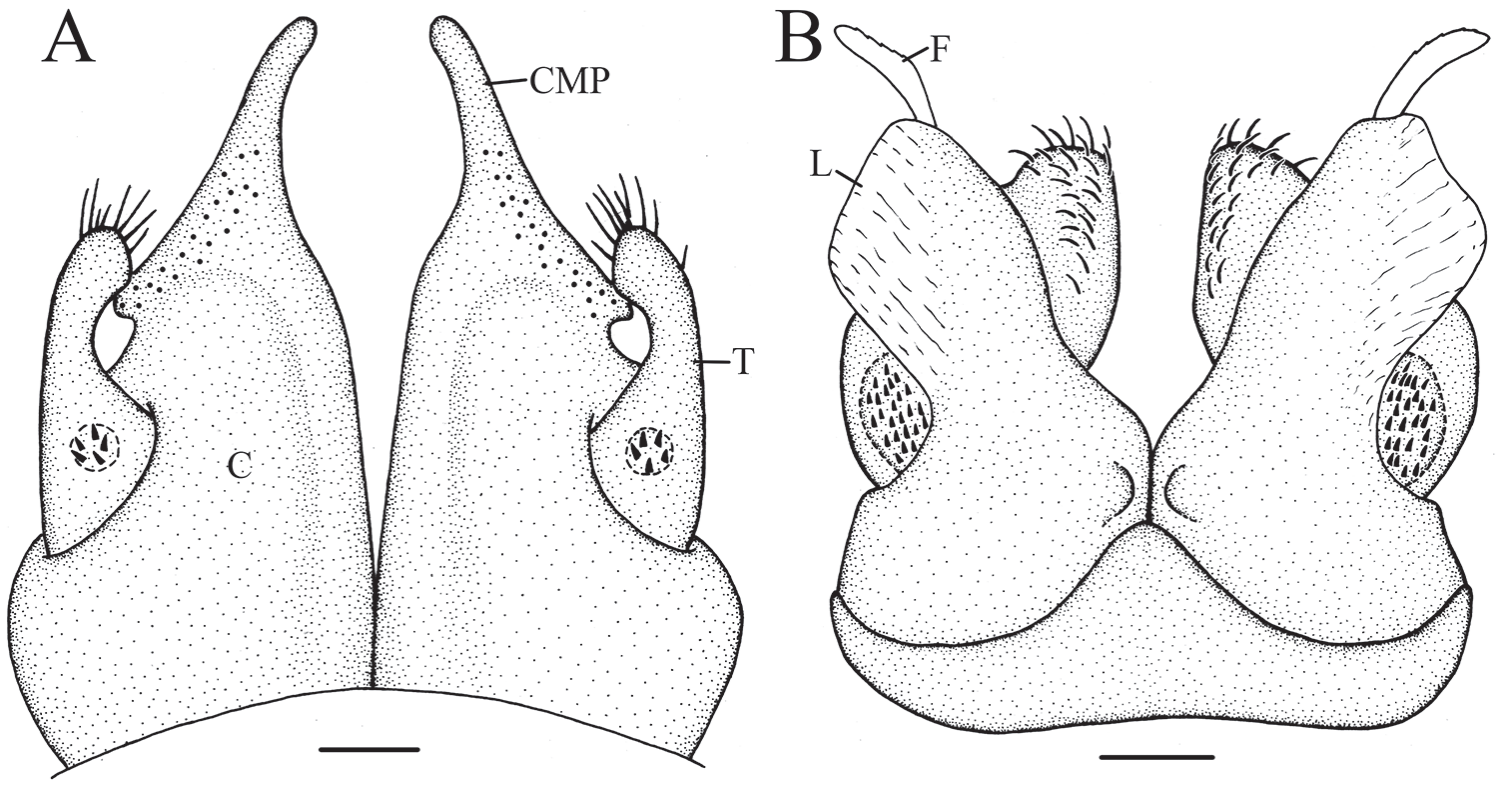
*Glyphiulus impletus* sp. n., holotype. **A** anterior gonopods, caudal view **B** posterior gonopods, caudal view. Abbreviations: C = coxosternum; CMP = coxosternal mesal process; F = flagellum; L = lamelliform lobe; T = telopodite. Scale bars: **A, B** 0.1 mm.

Figs 1D , 17 , 18 , 19 , 20 , 21

####### Type material.

**Holotype** male, China: Guangxi Zhuang Autonomous Region, Lingyun County, Luolou Town, Geding Village, Longcitun, Guanyin Cave 24°24.700'N, 106°49.517'E, alt. 830 m, 4 Jan. 2017, X.K. Jiang, H.M. Chen & X. Guo leg. (IBGAS). **Paratypes**: 22 males, 26 females and 9 juveniles, same date and locality as holotype (IBGAS); 17 males, 14 females and 43 juveniles, Lingyun County, Luolou Town, Geding Village, Longweitun, Paifang Cave 24°24.884'N, 106°48.900'E, alt. 830 m, 4 Jan. 2017, X.K. Jiang, H.M. Chen & X. Guo leg. (IBGAS).

####### Other material examined.

Seven males, 6 females and 4 juveniles, Lingyun County, Sicheng Town, Shuiyuan Cave 24°21.992'N, 106°34.670'E, alt. 450 m, 3 Jan. 2011, H.M. Chen leg. (IBGAS); 17 males and 14 females, Lingyun County, Sicheng Town, Naling Cave 24°21.926'N, 106°33.911'E, alt. 500 m, 4 Jan. 2011, H.M. Chen leg. (IBGAS); 3 males and 1 female, Fengshan County, Yuanyang Cave 24°32.518'N, 107°03.768'E, alt. 640 m, 3 Jan. 2017, X.K. Jiang, H.M. Chen & X. Guo leg. (IBGAS); 1 male, 2 females and 1 juvenile, Nandan County, Bachuan Cave 25°03.966'N, 107°37.392'E, 31 Jan. 2017, H.M. Chen & C. Chen leg. (IBGAS); 17 males, 24 females and 24 juveniles, Donglan County, Xinyan Village, Qiumotun, Ganma Cave 24°26.784'N, 107°20.584'E, alt. 320 m, 2 Feb. 2017, H.M. Chen & C. Chen leg. (IBGAS).

####### Etymology.

This specific name is derived from the Latin word *impletus*, meaning ‘plentiful’, referring to the large number of specimens of the new species in our collections.

####### Diagnosis.

The new species can be diagnosed by the following combination of morphological characteristics: (1) all crests on collum complete and fully developed, carinotaxic formula I–III + P + M; (2) telopodite of male leg I bi-segmented, shorter than coxal process; (3) coxosternal mesal process of anterior gonopod slender and strongly prolonged; (4) flagellum of posterior gonopod short and zigzag-shaped. See also Key below.

####### Description.

*Body* segments with 71–82p + 1a + T (holotype 73p + 1a + T). Body ca. 51–66 mm long and 2.3–3.2 mm wide (holotype 64 mm and 3.0 mm, respectively).

*Colouration*. Brown to dark brown *in vivo* (Fig. 1D). In fixed condition, yellow-brown to red-brown, tergal crests dark red-brown to castaneous brown (Fig. 17A–F).

*Head*. Each eye patch with 7–20 pigmented ocelli arranged in 1–3 irregular vertical rows (Fig. 17A, B). Antennae slender, 2.34–3.31 mm long. Terminal part of antennomeres V obviously expanded (Fig. 17B). Gnathochilarium with a separate promentum, polytrichous (Fig. 18A).

*Collum*. All crests on collum complete and fully developed, carinotaxic formula I–III + P + M (Fig. 17A, B).

*Body segments*. Postcollum constriction modest (Fig. 17A). Metaterga strongly crested (Fig. 17A–E). Metatergal crests divided into two transverse rows of tubercles, carinotaxic formula 2/2+I/i+3/3+I/i+2/2. Anterior tubercle (except ozoporiferous one) small and upright, posterior one directed caudally, both tubercles with sharp tips (Fig. 17A–E). Ozoporiferous tubercle round, higher than broad, obviously larger than other tubercles (Fig. 18E). Location of the tubercle behind ozopore relatively medial, set off from ozoporiferous tubercle in caudal view (Figs 17B–E, 18E). Lateral crests well developed. Midbody rings round in cross-section (Fig. 18E), 1.88–2.42 mm high (vertical diameter) and 1.91–2.42 mm wide (horizontal diameter), the ratio of height to width 0.95–1.00.

*Telson*. Epiproct simple, with a rounded caudal ridge and a strong dorsal tooth. Paraprocts convex, polytrichous. Hypoproct crescent-shaped (Fig. 17E, F).

*Walking legs*. Slender, 2.52–3.41 mm long, longer than body width (Fig. 18E, F).

*Male sexual characters*. Telopodite of male legs I strongly degraded, bi-segmented (Fig. 18B). Penes rather small and oval (Fig. 18C). Male legs II and III modified as usual (Fig. 18C, D). Femora VI and VII normal, not inflated.

*Anterior gonopods*. Coxosternum shield-like, sunken medially. Coxosternal mesal process slender and strongly prolonged. Telopodite thin, curved with a rounded tip, and a field of microsetae at base (Figs 19A, 20A, 21A).

*Posterior gonopods*. Mediolateral margins of coxite brush-like. Flagella short and zigzag-shaped. Lateral margin with a field of microsetae (Figs 19B, 20B, 21B).

####### Distribution.

Known from the type locality and several caves scattered in northwestern Guangxi.

**Figure 22.**
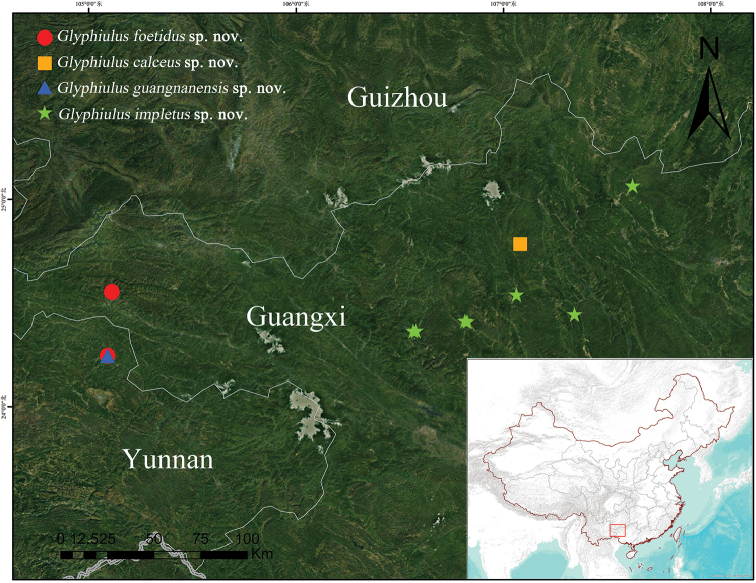
Distributions of *Glyphiulus foetidus* sp. n., *Glyphiulus calceus* sp. n., *Glyphiulus guangnanensis* sp. n. and *Glyphiulus impletus* sp. n.

### A key to species of the *Glyphiulus javanicus* group currently known from China (except *G. formosus*), based mainly on male characters

**Table:** 

1	Crests on collum fully developed (Figs 2A, 7A, 12A, 17A)	**2**
–	At least some crests on collum more or less obliterated	**12**
2	All crests on collum complete, carinotaxic formula of collum I–III + P + M (Figs 2A, 7A, 17A); epiproct with a strong dorsal tooth (Figs 2E, 7F, 17E); paraprocts convex (Figs 2F, 7E, 17F); telopodites of male legs I strongly reduced, 1–3-segmented (Figs 3B, 8B, 18B); flagella of posterior gonopods short (Figs 4B, 9B, 19B)	**3**
–	Not all crests on collum complete (Fig. 12A); epiproct with an axial dorsal rib (Fig. 12F); paraproct convex, with an evident depression near caudal edge (Fig. 12E); telopodites of male legs I normal or slightly reduced in size, 4–5-segmented (Fig. 13B); flagella of posterior gonopods long (Fig. 14B)	**8**
3	Coxosternal mesal processes of anterior gonopods elongated, obviously higher than telopodites (Figs 4A, 9A, 19A)	**4**
–	Coxosternal mesal processes of anterior gonopods short	**6**
4	Flagella of posterior gonopods with multiple branches (Fig. 5C)	***G. foetidus* sp. n.**
–	Flagella of posterior gonopods zigzag-shaped (Figs 10C, 20C)	**5**
5	Coxosternal mesal processes of anterior gonopods shoe-shaped (Figs 9A, 10A, 11A)	***G. calceus* sp. n.**
–	Coxosternal mesal processes of anterior gonopods thin and strongly elongated (Figs 19A, 20A, 21A)	***G. impletus* sp. n.**
6	Male femora VI and VII inflated	***G. recticullus***
–	Male femora VI and VII normal, not inflated	**7**
7	Telopodites of male legs I one-segmented; anterior gonopod coxosternum lower than telopodites	***G. pulcher***
–	Telopodites of male legs I bi-segmented; anterior gonopod coxosternum higher than telopodites	***G. echinoides***
8	Carinotaxic formula of collum 1a+2c+III–IV+5c+6a+pc+ma (Fig. 12A, B)	***G. guangnanensis* sp. n.**
–	Carinotaxic formula of collum not as above	**9**
9	Carinotaxic formula of collum I–III+4c+5a+pc+ma	**10**
–	Carinotaxic formula of collum I+2c+III–IV+5c+6a+pc+ma	**11**
10	Carinotaxic formula of midbody segments 2/2+I/i+3/3+I/i+2/2; coxosternal mesal processes of anterior gonopods broad	***G. latus***
–	Carinotaxic formula of midbody segments 2+I/i+3+I/i+2; coxosternal mesal processes of anterior gonopods slender	***G. paracostulifer***
11	Telopodites of male legs I normal, five-segmented; coxosternal mesal processes of anterior gonopods elongated and strong; lamelliform lobes of posterior gonopods obviously elongated	***G. intermedius***
–	Telopodites of male legs I reduced in size, five-segmented; coxosternal mesal processes of anterior gonopods slender; lamelliform lobes of posterior gonopods short	***G. liangshanensis***
12	Telopodites of male legs I normal; coxosternal mesal processes of anterior gonopods elongated; flagella of posterior gonopods long	***G. parobliteratus***
–	Telopodites of male legs I reduced in size, 4–5-segmented; coxosternal mesal processes of anterior gonopods not elongated; flagella of posterior gonopods absent	**13**
13	Collum not completely smooth, only medial crests obliterated	***G. zorzini***
–	Collum smooth, without apparent longitudinal crests	**14**
14	Coxosternal mesal processes of anterior gonopods folded	***G. obliteratoides***
–	Coxosternal mesal processes of anterior gonopods not folded	**15**
15	Carinotaxic formula of midbody segments 1/1+I/i+3+I/i+1/1; telopodites of male legs I with a claw	***G. obliteratus***
–	Carinotaxic formula of midbody segments 2/2+I/i+3/3+I/i+2/2; telopodites of male legs I without claw	**16**
16	Lamelliform lobes of posterior gonopods elongated	***G. sinensis***
–	Lamelliform lobes of posterior gonopods short	***G. subobliteratus***

## Supplementary Material

XML Treatment for Glyphiulus foetidus

XML Treatment for Glyphiulus calceus

XML Treatment for Glyphiulus guangnanensis

XML Treatment for Glyphiulus impletus
